# Neurogenetic disorders associated with mutations in the FERRY complex: a novel disease class?

**DOI:** 10.1242/bio.061808

**Published:** 2025-03-10

**Authors:** R. Madison Riffe, Gerald B. Downes

**Affiliations:** ^1^ Neuroscience and Behavior Graduate Program, University of Massachusetts Amherst, Amherst, MA, 01003, USA; ^2^ Biology Department, University of Massachusetts Amherst, Amherst, MA, 01003, USA; ^3^ Molecular and Cellular Biology Graduate Program, University of Massachusetts Amherst, Amherst, MA, 01003, USA

**Keywords:** FERRY complex, Neurogenetic disease, TBCK, PPP1R21, FERRY3, Rab5

## Abstract

The five-subunit endosomal Rab5 and RNA/ribose intermediary (FERRY) complex is a newly described protein complex consisting of TBCK, PPP1R21, FERRY3 (previously C12orf4), CRYZL1, and GATD1. The FERRY complex is proposed to function as a Rab5 effector to shuttle mRNA to the cell periphery for local translation, a process especially important in cells with far reaching processes. Interestingly, three members of the FERRY complex are associated with ultra-rare neurogenetic disorders. Mutation of TBCK causes TBCK syndrome, mutation of PPP1R21 is associated with PPP1R21-related intellectual disability, and mutation of FERRY3 results in an autosomal recessive intellectual disability. Neurologic disorders have yet to be associated with mutation of GATD1 or CRYZL1. Here, we provide a review of each FERRY complex-related neurologic disorder and draw clinical comparisons between the disease states. We also discuss data from the current cellular and animal models available to study these disorders, which is notably disparate and scattered across different cell types and systems. Taken together, we explore the possibility that these three diseases may represent one shared disease class, which could be further understood by combining and comparing known information about each individual disease. If true, this could have substantial implications on our understanding of the cellular role of the FERRY complex and on treatment strategies for affected individuals, allowing researchers, clinicians, and patient organizations to maximize the utility of research efforts and resources to support patients with these disorders.

## Teaser

**Summary:** This Review provides a summary of cellular and molecular data about *TBCK*, *PPP1R21*, and *FERRY3*, components of the FERRY complex, and compares clinical symptoms observed upon their mutation.

## Introduction

**Fig. 1.**
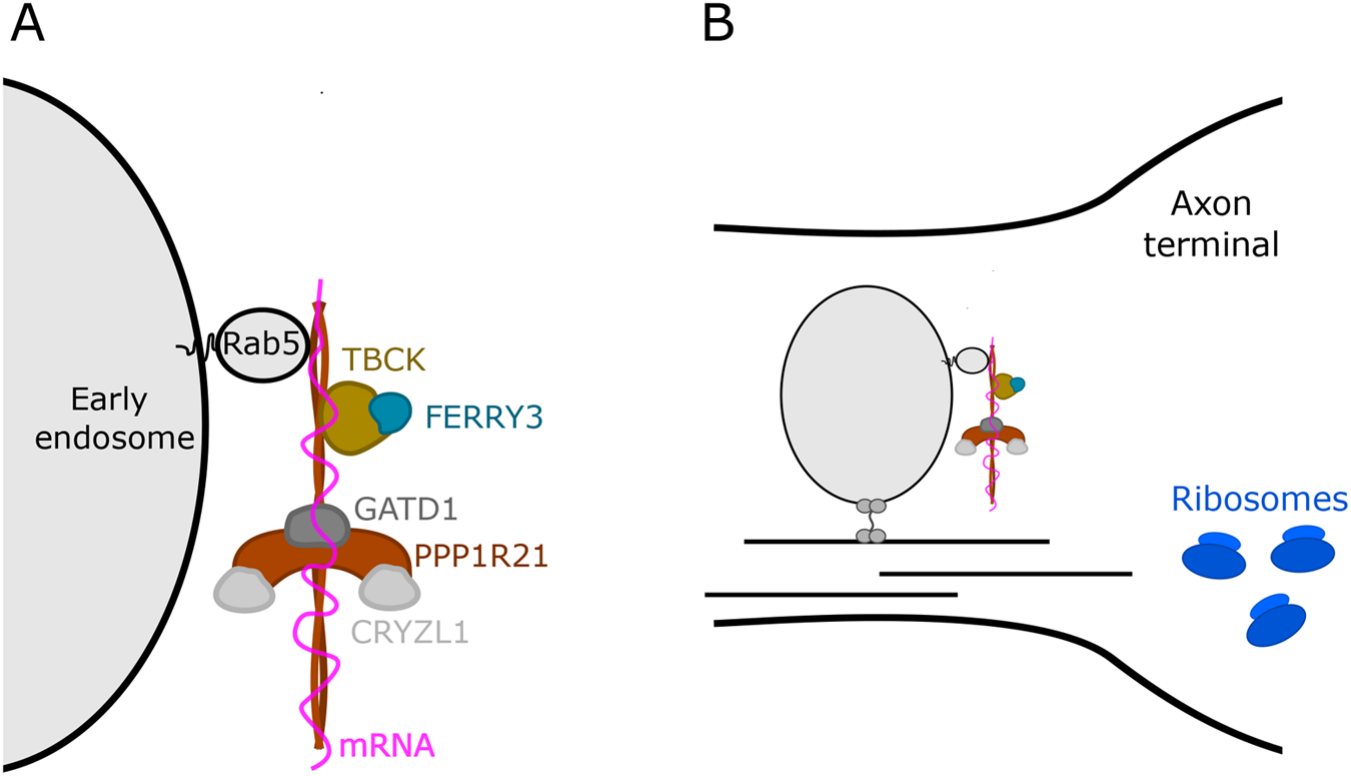
**Proposed structure and function of the FERRY complex.** (A) Cryo-EM data have resolved the majority of the FERRY complex structure. PPP1R21 serves as the backbone of the FERRY complex and directly binds active Rab5 on early endosomes. TBCK and FERRY3 were unable to be resolved via cryo-EM, but their binding location on PPP1R21 has been determined. The FERRY complex is proposed to bind mRNA via a complex binding interface consisting mostly of the PPP1R21 coiled-coils (Quentin et al., 2023; Schuhmacher et al., 2023). (B) The FERRY complex is proposed to shuttle mRNA to the cell periphery for local translation, a process especially important in cells with far-reaching processes, like neurons (Schuhmacher et al., 2023).

The Rab family of small G-proteins has important roles in vesicle trafficking. Rab5 binds early endosomes and regulates endocytosis through interaction with effector proteins, facilitating processes such as vesicle fusion and endosome maturation (Murray et al., 2002; Stenmark et al., 1994; Yuan and Song, 2020). The five-subunit endosomal Rab5 and RNA/ribose intermediary (FERRY) complex is a newly described protein complex proposed to function as a Rab5 effector (Quentin et al., 2023; Schuhmacher et al., 2023). The members of the FERRY complex are TBCK, PPP1R21, FERRY3 (previously known as C12orf4), CRYZL1, and GATD1, which appear in the complex at a stoichiometry 1:2:1:2:4, respectively. The backbone of the FERRY complex is formed by PPP1R21, which binds Rab5 on early endosomes when Rab5 is active and bound to GTP (Quentin et al., 2023; Schuhmacher et al., 2023). Structural analysis reveals that FERRY is unlike other described Rab5 effectors or RNA binding proteins in that it features an elongated protein complex, with PPP1R21 coiled-coils extending vertically from a central clamp-like structure (Quentin et al., 2023) (Fig. 1A).

After binding Rab5, the FERRY complex has been proposed to bind specific mRNAs and shuttle those mRNAs on the outside of early endosomes to local translation machinery in the cell periphery (Schuhmacher et al., 2023) (Fig. 1B). Local translation of proteins is essential for a rapid response to extracellular cues and is thought to be especially important in cells with long processes that extend far from the cell body, such as neurons (Holt et al., 2019; Turner-Bridger et al., 2020).

Notably, mutations in three different members of the FERRY complex (*TBCK*, *PPP1R21*, and *FERRY3*) are associated with rare neurologic disorders in human patients. Mutation of *TBCK* is associated with global developmental delay, hypotonia, and seizures (Bhoj et al., 2016; Chong et al., 2016; Guerreiro et al., 2016). Mutation of *PPP1R21* is associated with global developmental delay and hypotonia (Loddo et al., 2020; Rehman et al., 2019; Suleiman et al., 2018). Mutation of *FERRY3* is associated with intellectual disability and behavioral problems, with some reports of infantile hypotonia (Alazami et al., 2015; Hancarova et al., 2019; Philips et al., 2017; Rashvand et al., 2022). Mutation of the remaining two members of the FERRY complex, *glutamine amidotransferase-like class 1 domain containing 1* (*GATD1*) and *crystallin, zeta (quinone reductase)-like 1* (*CRYZL1*) has not yet been associated with neurologic disease.

The papers originally describing the FERRY Complex note that three FERRY Complex genes are associated with disease, but an in-depth comparison of the disease states has yet to be made (Quentin et al., 2023; Schuhmacher et al., 2023). Here, we will provide a summary of each of the three neurologic diseases associated with mutations in FERRY complex members. Then, we will draw comparisons between each of the disease states. We hope to highlight that while they are not completely identical, many similarities exist between these three disorders. If these disorders do share some cellular mechanisms of pathogenesis through the FERRY complex, which has yet to be determined, there could be a clear clinical advantage to considering them as one disease class.

## Summary of TBCK syndrome (OMIM #616900) and known function of TBCK

Information about TBCK syndrome comes to us primarily from clinical reports. Of the three disorders noted here, TBCK syndrome has been described most extensively in the literature and has the most currently identified cases. As the best described disorder, we also know more about the cellular consequence of TBCK disruptions, derived primarily from patient-derived cells. Some work has also been done to characterize TBCK disruptions in mice and zebrafish. Mutations in TBCK were first associated with a neurological disorder in 2015 (Alazami et al., 2015). The disease was fully described in 2016 and has now been diagnosed in over 100 children and young adults (https://www.tbckfoundation.org/). Unfortunately, there is no cure for TBCK syndrome, and the disease is often ultimately fatal.

TBCK syndrome patients demonstrate a wide range of clinical symptoms with a variable presentation reported from patient to patient (full scope of clinical features associated with TBCK syndrome shown in File S1). That said, there is still a collection of hallmark features that appear in many patients. TBCK syndrome is characterized by extremely low muscle tone, drug-resistant epilepsy, and global developmental delay (Bhoj et al., 2016; Chong et al., 2016; Guerreiro et al., 2016; Zapata-Aldana et al., 2019). TBCK syndrome patients often demonstrate reduced white matter volume and brain atrophy (Beck-Wödl et al., 2018; Ortiz-González et al., 2018; Zapata-Aldana et al., 2019). The identification of accumulations of intracellular storage material in neurons and glia post-mortem lead TBCK syndrome to be proposed as a novel lysosomal storage disorder, a class of diseases characterized by the inappropriate accumulation of lysosomal degradation products (Beck-Wödl et al., 2018). The clinical manifestations seen in TBCK syndrome patients is further reviewed in Durham et al. (2023).

TBCK syndrome is caused by loss-of-function mutations in *TBC1 domain containing kinase* (*TBCK*). *TBCK* encodes a highly conserved protein consisting of kinase-like, Tre2, Bub2, Cdc16 (TBC), and rhodanese homology domains. The kinase-like domain is missing a key residue found in functional kinases, suggesting that it is catalytically inactive (Liu et al., 2013). TBC domains are commonly found in Rab-GTPase activating proteins (Rab-GAPs) (Fukuda, 2011); however, no work has been done to determine if the TBC domain in TBCK functions as a Rab-GAP. Interestingly, despite the presence of a TBC domain in TBCK, within the FERRY complex, only PPP1R21 has been shown to directly bind Rab5 (Schuhmacher et al., 2023).

An appreciable amount of work has been performed on patient-derived TBCK-deficient cells and on engineered TBCK-deficient cells. Despite in-depth characteristics of several cell lines, the exact cellular role of TBCK is still unclear. TBCK syndrome has been associated with a host of lysosomal defects, including an increase in the number and size of lysosomes and a reduction in lysosomal proteolytic activity in patient-derived fibroblasts. TBCK has also been proposed to regulate the mechanistic target of rapamycin (mTOR) signaling pathway (Bhoj et al., 2016; Liu et al., 2013), a master regulator of cell growth, protein synthesis, proliferation, and autophagy (Brunn et al., 1997; Düvel et al., 2010; Kim et al., 2011). mTOR signaling is accomplished through two distinct but interconnected pathways mediated by mechanistic target of rapamycin complex 1 (mTORC1) and mechanistic target of rapamycin complex 2 (mTORC2).

Although many databases and published works cite that TBCK regulates the mTOR pathway, the relationship between TBCK and mTOR in the literature is unclear and seemingly conflicting results have been observed even within the same cell type (Table 1). An early TBCK report first demonstrated a reduction in mTORC1 signaling following RNAi disruption of TBCK in HEK293 cells (Liu et al., 2013). One of the first TBCK syndrome patient papers identified a reduction of pS6 signaling, a commonly used marker for mTORC1 activation, in immortalized lymphoblastoid and fibroblast cell lines derived from patients (Bhoj et al., 2016). A recent preprint validated the mTORC1 reduction seen in patient-derived fibroblasts (Angireddy et al., 2024 preprint). Finally, patient-derived induced neural progenitor cells (iNPCs) show reductions in mTORC1 signaling only after being stressed by the removal of EGF/FGF-2 from their growth media (Moreira et al., 2022).

**Table 1.** Regulation of mTOR signaling by TBCK is likely indirect and cell-type specific

Cell or tissue type	TBCK manipulation	Nutrient availability	Readout	Change	Implication	Reference
Hek 293	RNAi knockdown	Normal	mTOR ^i,q,w^	Decreased	Reduced mTORC1 and/or mTORC2 signaling	(Liu et al., 2013)
			p-mTOR ^w^			
			RPTOR ^q,w^			
			RICTOR ^q,w^			
			MLST8 ^q,w^			
			p-4EBP1 (Thr37/46) ^q,w^			
			p-P70S6K (Thr389) ^w^			
			4EBP1 ^q,w^			
			p-AKT(Ser473) ^w^			
			AKT ^q,w^	Unchanged		
			P70S6K ^q,w^			
Lymphocytes	Patient-derived	Normal	mTOR ^w^	Normal	Reduced mTORC1 signaling	(Bhoj et al., 2016)
			S6 ^w^	Normal		
			p-S6 (Ser235/236) ^w^	Decreased		
Fibroblasts	Patient-derived	Normal	p-S6 (Ser235/236) ^w^	Decreased	Reduced mTORC1 signaling	
iNPCs, derived from peripheral blood mononuclear cells	Patient-derived	Normal	p-S6 (Ser240/244) ^w^	Normal	Reduced mTORC1 signaling under starvation conditions	(Moreira et al., 2022)
		Restricted (EGF/FGF-2 removed)	p-S6 (Ser240/244) ^w^	Decreased		
Mouse brain	Heterozygous knockout mouse	Normal	S6 ^w^	Normal	Normal mTORC1 signaling	(Nair et al., 2023)
			p-S6 ^w^			
			4E-BP1 ^w^			
			p-4EBP1 (Thr37/46) ^w^			
			P70S6K ^w^			
			p-P70S6K (Thr389) ^w^			
HeLa	CRISPR-Cas9 generated InDel	Normal	RICTOR ^r^	Increased	Elevated mTORC2 signaling	(Schuhmacher et al., 2023)
			DEPTOR ^r^	Decreased	Reduced mTORC1 and/or mTORC2 signaling	
			P70S6K ^r^	Decreased		
			LAMTOR3 ^r^			
			RPTOR ^r^	Unchanged	Normal mTORC1 and/or mTORC2 signaling	
			4EBP1 ^r^			
			mTOR ^r^	Unchanged		
			MLST8 ^r^			
Raji	CRISPR-Cas9 generated InDel	Normal	p-S6 (Ser240) ^f^	Unchanged	Normal mTORC1 signaling	(von Beck and Jacob, 2023 preprint)
		Restricted (FBS removed)	p-S6 (Ser240) ^f^			
LP-1	CRISPR-Cas9 generated InDel	Normal	p-S6 (Ser240) ^f^	Unchanged	Normal mTORC1 signaling	
		Restricted (FBS removed)	p-S6 (Ser240) ^f^			
Caki-1	shRNA	Normal	pAKT ^w^	Decreased	Reduced mTORC2 signaling	(Park et al., 2023)
			AKT ^w^	Unchanged		
ReNcell VM (proliferating)	shRNA	Normal	p-S6 (Ser235/236) ^w^	Unchanged	Normal mTORC1 signaling	(Angireddy et al., 2024 preprint)
			S6 ^w^			
ReNcell VM (differentiated)	shRNA	Normal	p-S6 (Ser235/236) ^w^	Unchanged	Normal mTORC1 signaling	
			S6 ^w^			
HeLa	shRNA	Normal	p-S6 (Ser235/236) ^w^	Unchanged	Normal mTORC1 signaling	
Lymphoblasts	Patient-derived	Normal	p-S6 (Ser235/236) ^w^	Unchanged	Normal mTORC1 signaling	
Fibroblasts	Patient-derived	Normal	p-S6 (Ser235/236) ^w^	Decreased	Reduced mTORC1 signaling	

Superscript indicates assay, where f, flow cytometry; i, immunofluorescence; q, qRT-PCR; r, RNAseq; w, western blot. InDel, insertion and/or deletion.

Additional evidence towards a role for TBCK in mTORC1 signaling comes from autophagy defects seen in patient-derived cells. Autophagy is an output of reduced mTORC1 signaling that also relies on upstream input from the nutrient-sensing AMP-activated protein kinase (AMPK) pathway under starvation conditions (Kim et al., 2011). Patient-derived fibroblasts harboring a mutation known to result in a particularly severe form of TBCK syndrome show aberrant increases in LC3+ autophagosomes, autophagic flux, and mitophagy, which was interpreted as evidence of reduced mTORC1 signaling (Ortiz-González et al., 2018; Tintos-Hernández et al., 2021). In another study, patient-derived iNPCs show a normal number of LC3+ autophagosomes that are significantly smaller via immunostaining (Moreira et al., 2022), but it is unclear whether this reduction in autophagosome size indicates disrupted mTORC1 signaling. More recent work in HeLa cells with *TBCK* knocked down via shRNA shows increases in the number of LC3+ vesicles. They demonstrate that these vesicles are actually autolysosomes (autophagosomes that have fused with lysosomes), suggesting disruption of the clearance of autophagosomes (Angireddy et al., 2024 preprint).

While reductions in mTORC1 signaling have been observed in some systems following TBCK disruption, those reductions have not been observable in all systems. Despite patient-derived lymphoblasts demonstrating reductions in mTORC1 signaling in previous work (Bhoj et al., 2016), more recent work shows no change in mTORC1 signaling in the same cell type (Angireddy et al., 2024 preprint). TBCK knockdown in a B cell line and in LP-1 cells did not affect pS6 levels (von Beck and Jacob, 2023 preprint). In one of the FERRY papers, RNAseq of HeLa cells with mutated *TBCK* revealed some mTOR pathway components to be upregulated, some to be downregulated, and others to be unchanged (Schuhmacher et al., 2023). Lastly, a recently described *TBCK*^+/−^ mouse model shows no genotype-specific differences in mTORC1 signaling in total brain lysates (Nair et al., 2023). Thus, in the face of these conflicting data, the relationship between mTORC1 and *TBCK* still remains unclear.

There are also data to suggest that TBCK may regulate mTORC2, a regulator of cell proliferation. Reductions in TBCK have resulted in decreases in cell proliferation in HEK293 cells, patient-derived iNPCs differentiated into 3D neurospheres, and in neural progenitor ReNcells (Angireddy et al., 2024 preprint; Liu et al., 2013; Moreira et al., 2022). This proliferation reduction was shown to specifically affect dopaminergic neurons following shRNA knockdown of TBCK in ReNcells (Angireddy et al., 2024 preprint). TBCK knockdown via shRNA in a renal cancer cell line results in decreased mTORC2 signaling, as evidenced by decreased pAKT (Park et al., 2023). A comprehensive list of the expression of mTOR components and mTOR pathway direct outputs following disruption of *TBCK* is shown in Table 1. Together, these studies suggest TBCK's regulation of mTOR signaling may be indirect and cell-type specific. Future studies on TBCK and mTOR should consider these seemingly conflicting data.

Few studies have characterized *TBCK* deficient animal models, which highlights a considerable research gap in the field. Recent work has described a mouse line heterozygous for a *TBCK* knockout mutation (*TBCK*^+/−^) (Nair et al., 2023). Notably, on a C57BL/6J background, mice homozygous for the allele are embryonic lethal. Overall, *TBCK*^+/−^ mice display behavioral phenotypes indicative of reduced exploratory drive. They also report sex-specific behavioral phenotypes within the line. Male *TBCK*^+/−^ mice display a reduction in rearing during the open field test and reduced number of arm entries in the Y-maze test. Female *TBCK*^+/−^ mice have disruptions in the acoustic startle response and travel shorter distances on an elevated zero maze. Regardless of sex, *TBCK*^+/−^ mice also show disruptions in prepulse inhibition (Nair et al., 2023). Work to characterize the consequence of TBCK disruption in a whole organism has also been performed using zebrafish. *tbck* was identified as a gene required for proper heart development in a zebrafish screen. Knockdown of *tbck* expression using splice-blocking Morpholino resulted in zebrafish larvae with atrioventricular canal and heart looping defects, in addition to a smaller head, unabsorbed yolk, and pericardial edema (Ma et al., 2023). No neurological phenotypes were explored as part of that study.

## Summary of PPP1R21-related intellectual disability (OMIM #619383) and known function of PPP1R21

Compared to TBCK, less is known about the physiological roles of protein phosphatase 1 regulatory subunit 21 (PPP1R21). Most information comes from case reports of patients with PPP1R21-related intellectual disability. Mutation of this gene was first associated with intellectual disability in 2017 (Anazi et al., 2017). *PPP1R21* encodes a protein with conserved KLRAQ and TTKRSYEDQ domains (Rehman et al., 2019). The structure of PPP1R21 within the FERRY complex has been described in detail (Quentin et al., 2023). Patients with mutations in *PPP1R21* commonly experience global developmental delay, hypotonia, respiratory and feeding difficulties, and dysmorphic facial features. MRI abnormalities seen in patients include thinning of the corpus callosum, white matter abnormalities, cavum septum pellucidum, cerebellar vermis hypoplasia, and ventricular abnormalities (Anazi et al., 2017; Rehman et al., 2019; Suleiman et al., 2018). Less common symptoms include seizures, scoliosis, and ataxia and gait abnormalities (Almannai et al., 2024; Loddo et al., 2020; Rehman et al., 2019).

Most of the cellular characterization of PPP1R21 comes from two different studies of patient-derived fibroblasts. The staining pattern of PPP1R21 was noted to mimic that of vesicles in hTERT RPE-1 retinal epithelial cells, prompting researchers to examine the colocalization of PPP1R21 with various organelle markers. In those cells, PPP1R21 demonstrates nearly complete colocalization with early endosome antigen 1 (EEA1), but no colocalization with Golgi markers (Rehman et al., 2019). EEA1 staining is unchanged in PPP1R21 patient-derived fibroblasts, suggesting that PPP1R21 is not necessary for proper EEA1 localization. In those same fibroblasts, cells are able to uptake transferrin-488 properly, but clearance of transferrin is delayed, evident of endo-lysosomal dysfunction (Rehman et al., 2019). Patient-derived fibroblasts demonstrate a host of differentially expressed proteins. Those cells have elevated proteasome activity and reduced cell viability (Hentschel et al., 2023). Patient-derived fibroblasts also demonstrate aggregations of myelin-like bodies, reminiscent of cellular phenotypes seen in patients with lysosomal storage disorders (Hentschel et al., 2023; Rehman et al., 2019).

The only published animal model available to study PPP1R21 is a recently described zebrafish line. *Ppp1r21*-deficient zebrafish harboring a nonsense point mutation were characterized as part of a forward genetic screen to identify genes required for proper bile duct formation (Wu et al., 2023). These larvae die by 15 days post fertilization and have notable bile duct, gallbladder, and liver disruptions; however, no neurological phenotypes were examined in that study. Interestingly, the authors report aberrant increases in mTORC1 and mTORC2 signaling in the liver, as evidenced by immunostaining for pS6 and pAKT. When *ppp1r21* larvae were treated with mTOR pathway inhibitors, the bile duct phenotype was rescued, suggesting mTOR hyperactivity could lead to the pathogenesis of *ppp1r21* zebrafish (Wu et al., 2023).

## Summary of FERRY3 autosomal recessive intellectual disability (OMIM #618221) and known function of FERRY3

Of the three disorders discussed here, the least is known about FERRY3 autosomal recessive intellectual disability. Until recently, *FERRY3* was referred to as *C12orf4*, but major genomic databases currently agree on the official name *FERRY3*, which we will continue to use here. Evidence of the role of FERRY3 comes almost entirely from case reports of patients. In 2015, a study seeking to identify the causative gene mutations in a large cohort of 143 families with neurological disorders identified both *TBCK* and *FERRY3* as putative novel disease genes, with *FERRY3* mutation associated with global developmental delay (Alazami et al., 2015). A p.Leu328Pro mutation has been identified as a founder effect in northeastern Finland, associated with intellectual disability, delayed speech development, and neuropsychiatric symptoms (Philips et al., 2017). Other reported symptoms associated with *FERRY3* mutations include autism spectrum disorders, gait abnormalities, mild dysmorphic features, and hypotonia (Hancarova et al., 2019; Maddirevula et al., 2019; Philips et al., 2017). *FERRY3* mutations are also associated with a range of behavioral abnormalities such as aggressiveness and hyperactivity (Rashvand et al., 2022). The inheritance pattern of all published pathogenic *FERRY3* mutations is autosomal recessive.

To our knowledge, no studies have been conducted on FERRY3 patient-derived cells and very little work has been done to characterize the function of the FERRY3 protein. Work on the rat homologue of FERRY3 identified that it localized to the cytoplasm and has a role regulating mast cell activation (Mazuc et al., 2014). Lastly, bioinformatic analysis of FERRY3 suggests that it contains a conserved macro domain and may have a role in ADP-ribosylation, a post-translational modification where an ADP-ribose is added to a protein (Dudkiewicz and Pawłowski, 2019).

## Comparison of clinical features demonstrates notable, but not perfect, clinical overlaps

**Fig. 2.**
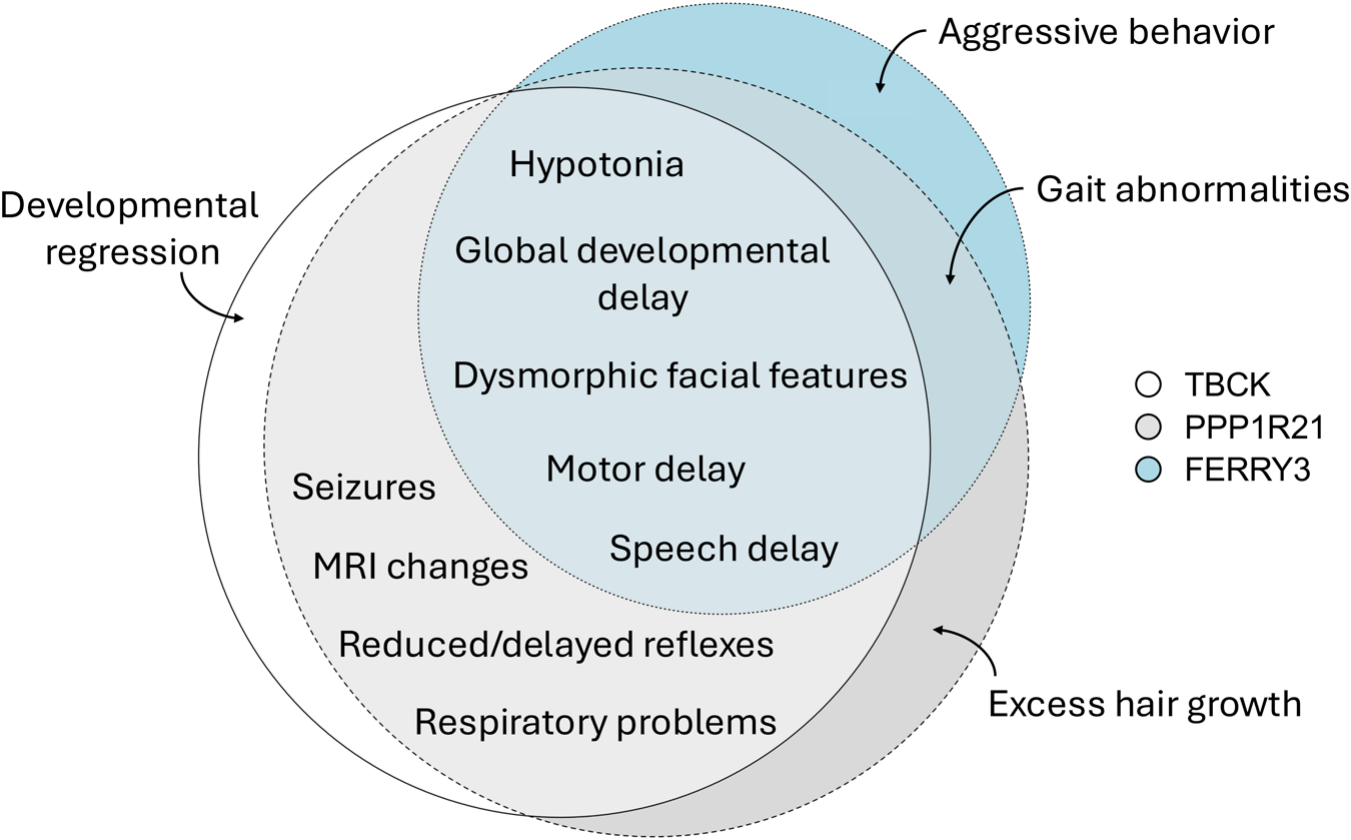
**Distribution of overlapping clinical features.** All features shown are present in at least 10% of the published patient cases for each disorder. Euler diagram created using eulerr R Shiny app (Larsson, 2021).

Because these three proteins share a proposed cellular mechanism, comparing the disorders associated with mutation of these genes could reveal important information about their possible shared cellular etiology. Given the rarity of each disorder and that different clinicians may report cases differently, comparing these disorders is challenging. For this Review, we have only included phenotypes described in published works. A detailed breakdown of each patient reported is shown in File S1. A comparison of the incidence of key clinical features for each of the three disorders is shown in Table 2. All three disorders are marked by speech and motor delays, hypotonia, and dysmorphic facial features. Of the three disorders, TBCK syndrome and PPP1R21-related intellectual disability share the most similar clinical features (Fig. 2). Only TBCK syndrome and PPP1R21-related intellectual disability share the presence of seizures, reduced reflexes, feeding and respiratory difficulties, and notable MRI characteristics, including white matter and ventricular abnormalities. Only PPP1R21 and FERRY3 patients share the reported presence of ataxia and gait abnormalities. The vast majority of cases of all three disorders are caused by homozygous variants inherited in an autosomal recessive pattern, with a few cases of pathogenic compound heterozygous cases reported in TBCK (Bhoj et al., 2016).

**Table 2.** Incidence of clinical features seen in TBCK syndrome, PPP1R21-related intellectual disability, and FERRY3 autosomal recessive intellectual disability

Clinical feature	TBCK	PPP1R21	FERRY3
Global developmental delay	+++	+++	+
Speech delay	++	+	+++
Motor delay	++	+	+
Developmental regression	+		
Hypotonia	+++	+++	+
Reduced or delayed reflexes	++	++	
Dysmorphic facial features	+++	+++	+
Seizures	+++	+	
Ataxia/gait abnormalities		+	+
Scoliosis	+	+	
Respiratory difficulties	+	++	
Feeding difficulties	+	+++	
Abnormal white matter (MRI)	++	++	
Ventricle abnormalities (MRI)	+	++	
Thin/dysmorphic corpus callosum (MRI)	+	++	
Cavum septum pellucidum (MRI)		+	
Cerebellar vermis hypoplasia (MRI)		+	
Autism spectrum disorder			+
Aggressive behavior			+
Excess hair growth		++	

Incidence was derived from taking the number of occurrences of a phenotype and dividing that by the total number of patients. +, 10-40% incidence; ++, 41-60% incidence; +++, >61% incidence across all reported cases. Total number of patients reviewed: 66 for TBCK; 24 for PPP1R21; 16 for FERRY3. Citations associated with the reported incidence of each feature are included in supplemental File 1.

Developmental regression and a progressive disease phenotype are hallmarks of TBCK syndrome. One case report of an adult patient with PPP1R21-related intellectual disability describes the patient's facial dysmorphism worsening with age (Loddo et al., 2020). This report emphasizes that, as *PPP1R21* mutations have been shown to result in cellular accumulations that likely worsen with time, a progressive disease phenotype could be expected in *PPP1R21* patients (Loddo et al., 2020). To our knowledge, no progressive phenotypes have been reported for patients with mutations in *FERRY3*. Ultimately, additional longitudinal studies are needed to evaluate the progressive nature of the disorders caused by mutations in *PPP1R21* or in *FERRY3*.

In reviewing this literature, we note that “milder” behavioral features seem to be underreported in TBCK syndrome compared to the less severe FERRY3 autosomal recessive intellectual disability. For example, hand biting behavior is noted in the literature for 12.5% of FERRY3 patients (File S1). To our knowledge, no case of hand biting behavior is published for TBCK syndrome. However, from personal correspondence with TBCK families through the TBCK Foundation, we know that hand biting represents a common challenge among TBCK patients. Thus, the full scope of the behavioral manifestations in at least TBCK syndrome seems to be underreported in the literature.

We acknowledge that some of the key clinical features described here are likely shared across many different, unrelated neurodevelopmental disorders. However, the presence of a shared proposed cellular mechanism between these three proteins makes it especially important to consider the potential for a shared pathological mechanism.

It is also worth noting that several germline and somatic variants of FERRY complex members are associated with tumors and cancers. In patients with hepatocellular carcinoma, sequencing of circular tumor DNA revealed the presence of frameshift-inducing insertional mutations in *TBCK* (Gao et al., 2021). An additional connection to *TBCK* and tumor development comes from a *TBCK*-*P4HA2* gene fusion detected in soft tissue angiofibroma samples that leads to a putative fusion protein with unknown cellular consequences (Panagopoulos et al., 2016). In a genome-wide association study (GWAS) of people of Asian descent, colorectal cancer was significantly associated with an intronic variant in *PPP1R21* (Lu et al., 2018)*.* In addition, a GWAS study revealed a variant in *PPP1R21* that is significantly associated with breast cancer in women defined as obese (Ponomarenko et al., 2024). Genome-wide variants in *GATD1* resulting in an active gene promoter and enhancer are associated with breast cancer (Xavier et al., 2024). Finally, within a group of cervical cancer tumor samples, differential methylation of *CRYZL1* was associated with cervical cancer survival risk (Zhang et al., 2021). The overlap between genes associated with cancers and with neurodegenerative diseases has been noted and discussed, as reviewed in (Houck et al., 2019). We point out these associations to highlight that better understanding the cellular roles of these genes has important human health implications beyond just for neurogenetic disorders and to emphasize that much is still unknown about the cellular role of these genes.

## Additional cellular and animal models are needed to determine if these disorders share pathological mechanisms

Despite each of these genes being highly conserved from humans to nonhuman primates, mouse, zebrafish, *Drosophila*, and *C. elegans* (Schuhmacher et al., 2023), there are very few animal models established for these disorders. Further, beyond the work done in the FERRY complex papers (Quentin et al., 2023; Schuhmacher et al., 2023), the experiments that have been performed on cellular and animal models for each disorder are notably disparate. The hypothesis we explore here – that these three disorders may share pathogenic cellular mechanisms – is testable. In order to truly evaluate if these disorders share similar cellular pathogenesis, additional experiments need to be conducted in the same cellular and animal models. That said, the experiments that have been performed do provide some insight into their potential shared etiology.

Most of the comparisons between these disorders at the cellular level come from the FERRY complex papers, but earlier reports do implicate both TBCK and PPP1R21 as regulators of endocytosis (Collinet et al., 2010; Rehman et al., 2019). Despite the FERRY complex binding directly to Rab5, a known regulator of endocytosis, a role for the FERRY complex in endocytosis has yet to be determined (Schuhmacher et al., 2023). Although TBCK and PPP1R21 have been implicated in the regulation of endocytosis, it is not clear whether this function is performed independent of the FERRY complex.

Both TBCK and PPP1R21-derived patient cells demonstrate inappropriate intracellular aggregations such as those seen in lysosomal storage disorders (Beck-Wödl et al., 2018; Hentschel et al., 2023; Rehman et al., 2019). Further, both TBCK and PPP1R21 have been implicated in mTOR signaling, although disruptions in TBCK have usually resulted in decreased or no changes in mTOR signaling (Table 1), while the *ppp1r21* zebrafish has aberrant increases in mTOR signaling (Wu et al., 2023). In a structural analysis of the FERRY complex, Quentin et al. explored the consequence of a pathogenic *PPP1R21* mutation that results in PPP1R21 being truncated by 84 amino acids. They conclude that this mutation would eliminate the binding site for TBCK and FERRY3, resulting in a FERRY complex that has only three of the five complex members, yet has no disruption to mRNA binding capabilities. Importantly, following truncation of PPP1R21 that abolished TBCK and FERRY3 binding, the FERRY complex was also unable to bind Rab5 (Quentin et al., 2023). This suggests that presence of TBCK and FERRY3 are necessary for FERRY binding of Rab5 in humans, providing a possible pathogenic mechanism that these three disorders could share.

Following knockout of either *TBCK*, *PPP1R21*, *CRYZL1*, or *GATD1* in HeLa cells, knockout of *TBCK* had the most substantial impact on FERRY complex localization, as indicated by the colocalization of early-endosomes and mRNA. RNAseq on each of those knockout cell lines revealed unique profiles of differentially expressed genes (Schuhmacher et al., 2023). While these sequencing results suggest that knockout of different complex members could differentially affect overall FERRY complex function, it is also possible that members of the FERRY complex have additional, non-overlapping cellular roles outside of their role in the FERRY complex. Further, it is not clear whether dysfunction in the proposed mRNA trafficking mechanism of the FERRY complex could cause neurologic disease, or if disruption of some other FERRY complex function is pathogenic. Mutations in *RAB5C* are known to cause a disorder characterized by developmental delay and macrocephaly (Koop et al., 2023). Thus, it is a distinct possibility that dysregulation of Rab5 trafficking could be the pathogenic mechanism underlying mutation of FERRY complex disease genes.

## Conclusions

Here, we have described three rare neurologic disorders resulting from mutations in either *TBCK*, *PPP1R21*, or *FERRY3*. Importantly, these three genes encode proteins that share a proposed cellular mechanism through the FERRY complex (Quentin et al., 2023; Schuhmacher et al., 2023). Each of these three disorders share major clinical features, including global developmental delay, hypotonia, and dysmorphic facial features. Because of the deep unmet clinical need represented by each of these disorders and their potential shared cellular mechanism of pathogenicity, we encourage researchers to consider that these three disorders may represent one novel disease class.

In comparing each disorder described here, it is important to note that the number of published cases varies widely across the disorders. Our review identified 66 published descriptions of TBCK syndrome, 24 published descriptions for PPP1R21-related intellectual disability, and 16 published descriptions of FERRY3 autosomal recessive intellectual disability. Because of these low numbers, especially for FERRY3, one or two reports could dramatically skew the published incidence of clinical features for any of the disorders. Additionally, it is likely that all three of these disorders are vastly under-diagnosed and under-reported. For example, one TBCK syndrome case study points out that, with increasing access to genetic testing in Puerto Rico, they diagnosed four new TBCK patients within 6 months (De Luca-Ramirez et al., 2023). Thus, the incidence of all three of these disorders could be higher than the published numbers currently suggest, emphasizing the importance of increasing global access to genetic testing, as well as the need to better understand these disorders and their pathogenesis.

TBCK syndrome families and patients are supported by the work of the TBCK Foundation, a robust parent-led advocacy and support group (https://www.tbckfoundation.org/). To our knowledge, no patient advocacy groups have been established for patients with mutations in either *PPP1R21* or *FERRY3*. Further research into the pathogenic mechanisms of each disorder is imperative. Considering these disorders together could unify research and advocacy efforts, increasing patient support and facilitating the development of new therapies.

## Supplementary Material

10.1242/biolopen.061808_sup1 Supplementary information

File S1.
