# Supplementary material for: Neurogenetic disorders associated with mutations in the FERRY complex: a novel disease class?
Source: Biol Open. 2025 Mar 10;14(3):BIO061808. doi: 10.1242/bio.061808 (PMC11928052; doi:10.1242/bio.061808)
Supplement: Supplementary information [file biolopen-14-061808-s1.pdf]

File S1.

Available for download at
https://journals.biologists.com/bio/article-lookup/doi/10.1242/bio.061808#supplementary-data

Biology Open • Supplementary information
